# The World’s Columbian Dental Congress—Change of Time

**Published:** 1893-02

**Authors:** A. O. Hunt

**Affiliations:** Iowa City, Iowa


### Domestic Correspondence.

### THE WORLD'S COLUMBIAN DENTAL CONGRESS—CHANGE OF TIME.

To the Editor of the International Dental Journal:

Sir,—The following communication was received from President Bonney, of the World's Congress Auxiliary, which necessitates a change in the time of meeting, and also a rearrangement of the order of business for the World's Columbian Dental Congress.

"The Dental Congress has been assigned generally to the week commencing Monday, August 14, 1893. The Congresses of Science and Philosophy have been assigned to the week commencing Monday, August 21, 1893. With more than a hundred congresses to provide for, you will readily understand the extraordinary difficulty of making suitable arrangements for each, but the extra provision which has been made for the places of meeting will render practicable arrangements which, under the ordinary circumstances, would be simply impossible. When the congresses were first proposed we expected to have only one large audience-room with a suitable number of smaller halls; but as the World's Congress' work enlarged, the places of meeting were also made more adequate. As the World's Congress Art Palace is now planned, there will be two large audience-rooms capable of accommodating three thousand persons each, and more than twenty smaller halls which will seat from three hundred to seven hundred persons each; thus providing for no less than thirty-six large meetings, and three hundred and sixty smaller meetings in a single week, by holding morning, afternoon, and evening sessions. Among the other congresses assigned to be held in parallel with the Dental Congress are those of Pharmacy, Medical Jurisprudence, and Horticulture. For all these the accommodations will be adequate. You understand, of course, that everything in the nature of an exhibit is required by the Exposition authorities to go to Jackson Park. The congresses deal not with things, but with men; not with matter, but with mind."

In accordance with the above statement the time of meeting will be from Monday, August 14, to Saturday, August 19, inclusive.

Please note this change in your journal.

Yours very truly,

A. O. HUNT.

IOWA CITY, IOWA, January 16, 1893.

